# Isolation, Molecular Characterization, and Pathogenicity Evaluation of a Genotype GI Feline Calicivirus Isolate

**DOI:** 10.3390/microorganisms14091958

**Published:** 2026-09-04

**Authors:** Jia-You Xing, Wen-Jie Xu, Zi-Xuan Fu, Ying-Na Xu, Jing-Yang Li, Jiang Wang, Bei-Bei Chu, Lei Zeng, Sheng-Li Ming

**Affiliations:** 1 College of Veterinary Medicine, Henan Agricultural University, Zhengzhou 450046, China; 2 Key Laboratory of Animal Biochemistry and Nutrition, Ministry of Agriculture and Rural Affairs, Zhengzhou 450046, China; 3 Key Laboratory of Veterinary Biotechnology of Henan Province, Henan Agricultural University, Zhengzhou 450046, China; 4 Ministry of Education Key Laboratory for Animal Pathogens and Biosafety, Zhengzhou 450046, China; 5 Longhu Advanced Immunization Laboratory, Zhengzhou 450046, China; 6 International Joint Research Center of National Animal Immunology, Henan Agricultural University, Zhengzhou 450046, China

**Keywords:** feline calicivirus, genotype GI, virus isolation, molecular characterization, pathogenicity, phylogenetic analysis, VP1

## Abstract

Feline calicivirus (FCV) is a highly heterogeneous pathogen and a major cause of feline upper respiratory tract disease, highlighting the need for continuous surveillance of its genetic diversity and pathogenic characteristics. In this study, we isolated and comprehensively characterized a newly isolated FCV strain, HN/ZZ/2025, from cats at a feline trading market in Zhengzhou, China, and systematically evaluated its molecular features, in vitro replication characteristics, physicochemical properties, and pathogenicity in cats. Phylogenetic analysis classified HN/ZZ/2025 as genotype GI. The isolate replicated efficiently in CRFK, F81, and Fc3Tg cells, reaching peak titers of 10^7.18^, 10^7.50^, and 10^5.89^ TCID_50_/0.1 mL, respectively, and exhibited typical calicivirus-like particles with diameters of 35–40 nm. Complete genome analysis showed that HN/ZZ/2025 shared the highest nucleotide and amino acid sequence identities of 85.7% and 62.8%, respectively, with the closely related Chinese FCV strain CH-JL4, and revealed distinct amino acid variations within the hypervariable E region of the VP1 capsid protein. Experimental infection of cats (n = 3/group) resulted in pyrexia, with rectal temperatures reaching 39.8–40.2 °C, weight loss, oral ulceration, and persistent viral RNA shedding from 1 to 21 days post-infection. Viral RNA and VP1 antigen were detected in the lungs, trachea, kidneys, and spleen, indicating systemic dissemination and broad tissue distribution. Although no mortality occurred during the observation period, the observed clinical signs, viral dissemination, and histopathological lesions indicate that HN/ZZ/2025 is capable of causing clinically evident disease in experimentally infected cats. These findings provide useful insights into the molecular epidemiology, tissue tropism, and host–virus interactions of circulating FCV strains and establish HN/ZZ/2025 as a useful isolate for future studies of FCV genetic diversity and pathogenesis.

## 1. Introduction

As a primary companion species, domestic cats (*Felis catus*) maintain intimate proximity to human populations, with a global population now exceeding 600 million [1]. Feline infectious diseases are of paramount importance. Upper respiratory tract diseases (URTDs) are frequently encountered in feline medicine and are characterized by a spectrum of clinical manifestations, including increased serous to mucopurulent ocular and nasal discharge, corneal ulceration, oral stomatitis, and pyrexia [2,3]. Among the primary etiological agents, feline calicivirus (FCV) is a major pathogen affecting both domestic and wild felids and has long been recognized as a leading cause of feline URTDs and oral ulceration [4,5,6,7]. In recent years, FCV has undergone continuous evolution, giving rise to diverse genotypes under immune selective pressure [8]. As a result, FCV strains demonstrate a remarkable capacity for escaping host immune clearance [8]. At present, no specific antiviral therapeutics are available for FCV, and control strategies rely primarily on vaccination. However, existing inactivated vaccines—typically targeting feline panleukopenia virus (FPV), FCV, and feline herpesvirus type 1 (FHV-1)—frequently offer incomplete protection against emerging FCV variants [9,10,11,12]. These findings underscore the necessity for continued investigation into FCV to better elucidate viral infection dynamics in companion animals [1].

FCV is a highly transmissible pathogen taxonomically classified within the genus Vesivirus of the family *Caliciviridae*. This family encompasses significant human pathogens, such as noroviruses, as well as several animal-specific viruses, including rabbit hemorrhagic disease virus and European brown hare syndrome virus. The designation *Caliciviridae* is derived from the characteristic chalice-shaped surface depressions observed on the virion architecture [13]. FCV is an RNA virus with a high mutation rate that is ubiquitously distributed among domestic feline populations [14]. While host-restricted to felids and lacking zoonotic potential, FCV exhibits extensive genetic and antigenic variability [15]. The virus is endemic in feline populations [16] and presents with diverse clinical signs, including URTDs, lingual ulceration, periodontitis, and limping syndrome. Over the past two decades, certain lineages have evolved into virulent systemic FCV (VS-FCV) variants characterized by enhanced replication kinetics. Clinical manifestations of VS-FCV can include severe alopecia, cutaneous ulceration, and necrotizing pododermatitis of the pinnae and nostrils, often accompanied by serous crusting. Additional systemic complications include subcutaneous edema, bronchointerstitial pneumonia, and multi-organ necrosis (pancreas, liver, and spleen), culminating in high mortality rates among infected individuals [13,17,18,19,20,21,22,23,24,25].

The FCV genome consists of a positive-sense, single-stranded RNA of approximately 7.5 kilobases, which facilitates rapid evolutionary adaptation [13]. The non-segmented genomic RNA contains three functional open reading frames (ORFs) encoding six non-structural proteins (NS1–NS6/7) and two structural proteins (VP1 and VP2) [26]. ORF1 encodes a large polyprotein that undergoes post-translational cleavage into non-structural components, such as protease and polymerase [27]. ORF2 encodes a polyprotein that is proteolytically processed to yield the leader of the capsid protein and the major capsid protein (VP1), while ORF3 encodes the minor capsid protein (VP2) [28]. The mature viral capsid is architecturally composed of 180 copies of VP1 and 12 copies of the low-abundance VP2 protein [29].

Since its initial isolation in 1957 from cats presenting with respiratory symptoms [30], FCV has demonstrated global ubiquity, with detections reported throughout Europe, the Americas, and Asia [31,32]. In 1992, the virus was identified in ulcerative oral lesions of African lions and Siberian tigers; subsequently [33], it was first isolated from tigers in China in 2000. These findings indicate that FCV is geographically widespread and possesses a broad host range, with nearly all felid species being susceptible to infection [34,35]. As an RNA virus, FCV is predisposed to frequent mutations, leading to the emergence of highly virulent variants. Among these, circulating strains of the GI genotype are associated with virulent systemic disease (VSD-FCV) [36]. While historically rare, these strains have disseminated globally following the characterization of VS-FCV in the United States in the 1990s [37]. The expansion of the feline breeding industry in China, combined with high population densities, has provided a fertile environment for viral propagation. The lack of robust preventive measures and the continuous mutation of the virus have compromised vaccine efficacy, posing a significant threat to the health and conservation of both domestic and wild felids [19]. Despite extensive studies on FCV molecular epidemiology and pathogenicity, important gaps remain in the characterization of recently circulating FCV strains in China. In particular, the genetic diversity of newly emerging isolates has not always been accompanied by systematic evaluation of their in vitro replication characteristics and in vivo pathogenicity. Moreover, the relationship between genomic variation, particularly changes within the hypervariable regions of the VP1 capsid protein, and the biological properties of circulating FCV strains remains incompletely understood. Therefore, continuous isolation and characterization of contemporary FCV strains from different geographic regions are necessary to improve our understanding of FCV evolution and pathogenic diversity.

In the present study, we isolated an FCV strain, designated HN/ZZ/2025, from a feline trading market in Zhengzhou, Henan Province, China. We characterized its complete genome and phylogenetic relationships and systematically evaluated its in vitro replication properties, physicochemical stability, and in vivo pathogenicity in cats. We further investigated viral shedding, tissue distribution, and histopathological changes following experimental infection. These data provide useful insights into the molecular epidemiological landscape, replication characteristics, and pathogenic dynamics of circulating FCV strains in China, providing a useful isolate for future studies of FCV genetic diversity and pathogenesis.

## 2. Materials and Methods

### 2.1. Ethics Approval and Consent to Participate

All experimental procedures involving animals were reviewed and approved by the Institutional Animal Care and Use Committee of Henan Agricultural University (Approval No. HNND2024050715). The study was conducted in accordance with relevant institutional regulations and animal welfare principles, including the 3Rs principles (Replacement, Reduction, and Refinement). The animals were monitored throughout the experimental period to minimize discomfort and distress. The scheduled experimental endpoint was 21 days post-infection, after which all animals were humanely euthanized by intravenous administration of pentobarbital sodium (100 mg/kg). The organs were subsequently collected for virological, Western blot, histopathological, and immunohistochemical analyses, and the remaining carcasses were disposed of according to institutional biosafety procedures.

### 2.2. Cells and Antibodies

CRFK (ATCC, CCL-94), F81 (Cellosaurus, CVCL_9259), and Fc3Tg (ATCC, CCL-176) cells were maintained in Eagle’s Minimum Essential Medium (EMEM, BNCC, Beijing, China) supplemented with 10% fetal bovine serum (FBS, Gibco, Thermo Fisher Scientific, Waltham, MA, USA) at 37 °C in a humidified 5% CO_2_ atmosphere. A rabbit polyclonal antibody (pAb) targeting the VP1 protein of FCV was generated and characterized in our laboratory. Primary antibodies against GAPDH (10494-1-AP), CoraLite488-conjugated Goat Anti-Rabbit IgG (H+L) (SA00013-2) and HRP-conjugated goat anti-rabbit IgG (SA00001-2) secondary antibodies were purchased from Proteintech (Wuhan, China).

### 2.3. Sample Collection and Detection

In January 2025, a total of 22 cats exhibiting clinical signs suggestive of FCV infection were selected for sampling at a feline trading market in Zhengzhou, Henan Province, China. Oral and nasal swabs were collected from each cat and combined as one clinical sample per cat, resulting in a total of 22 clinical samples. The sampling strategy was based on the presence of clinical manifestations suggestive of FCV infection. The samples were suspended in phosphate-buffered saline (PBS, pH 7.2) and transported to the laboratory under refrigerated conditions. Because the cats were obtained from a commercial feline trading market, detailed information regarding their age, sex, breed, vaccination history, and previous treatment status was not available or could not be reliably verified. Total viral DNA/RNA was extracted using the FastPure Viral DNA/RNA Mini Kit (Vazyme, RC311, Nanjing, China), and cDNA synthesis was performed using HiScript IV All-in-One Ultra RT SuperMix (Vazyme, R433-01). The presence of FCV, FPV, FHV, FIPV, FBoV, and FeAstV was determined by PCR using specific primers (Supplementary Table S1).

### 2.4. Virus Isolation, Cultivation and Purification

FCV-positive specimens were selected for viral isolation. Homogenates were vortexed and centrifuged at 12,000 rpm for 5 min at 4 °C. The resulting supernatant was filtered (0.22-μm, Biosharp, Beijing, China) and diluted 1:10 in serum-free MEM. Confluent CRFK cell monolayers were washed twice with PBS and inoculated with the suspension supplemented with 1% Penicillin-Streptomycin-Gentamicin (Solarbio, P1410, Beijing, China). Cells were monitored for cytopathic effects (CPE) and harvested after three freeze–thaw cycles. For purification, three consecutive rounds of plaque purification were conducted using a 1% low-melting-point agarose overlay (Solarbio, A8350). Visible plaques were identified via Neutral Red staining (Beyotime, C0125, Shanghai, China) and individual plaques were selected for clonal expansion and subsequent PCR verification.

### 2.5. Next-Generation Sequencing Analysis

Metaviral RNA libraries were constructed using the NEBNext Ultra II RNA Library Prep Kit (Illumina, New England Biolabs, Ipswich, MA, USA). Quality-filtered reads were aligned to the host reference genome using minimap2 (version v2.28) to eliminate host-derived contamination. De novo assembly of clean reads was performed using MEGAHIT (version v1.2.9), and ORFs were predicted via Prodigal (version v2.6.3). Functional annotation was conducted by aligning sequences against the Swiss-Prot database using MMseqs2 (version v2.0).

### 2.6. Genome Sequencing and Phylogeny Analysis

Full-length FCV genomic amplification was conducted using primers designed based on NGS results and NCBI reference sequences (Supplementary Table S2). The genome was amplified in three overlapping segments, cloned using the one-step ZTOPO-Blunt/TA kit (ZOMANBIO, Beijing Zoman Biotechnology Co., Ltd., Beijing, China), and subjected to Sanger sequencing. The FCV isolate, designated HN/ZZ/2025, was deposited in GenBank (Accession No. PX408781). Phylogenetic reconstruction was performed using the Maximum-likelihood (ML) method in MEGA version 7.0, based on the General Time Reversible (GTR+Γ+I) substitution model. Bootstrap values were calculated from 1000 replicates.

### 2.7. Virus Identification and Growth Curve

#### 2.7.1. Viral Propagation and Titration

The purified FCV strain HN/ZZ/2025 was inoculated onto confluent monolayers of CRFK, F81, and Fc3Tg cells in 60 mm culture dishes. CPE were monitored daily and documented using an inverted microscope (Olympus, Tokyo, Japan). Upon the appearance of approximately 80% CPE, the cultures were subjected to three freeze–thaw cycles. Cell debris was removed by centrifugation at 8000 rpm for 10 min at 4 °C, and the virus-containing supernatants were harvested and stored at −80 °C.

The 50% tissue culture infectious dose (TCID_50_) was determined using the Reed-Muench mathematical model. Briefly, cells were seeded in 96-well plates at a density of 1 × 10^4^ cells per well. After 24 h, the cells were inoculated with 10–fold serial dilutions of the virus (10^−1^–10^−12^) and incubated for 1 h at 37 °C. Following adsorption, the inoculum was replaced with 100 μL of maintenance medium (MEM supplemented with 2% FBS). The plates were incubated for 3–5 days and monitored daily for CPE. Each titration was performed in triplicate to calculate the mean viral titer.

#### 2.7.2. Immunoblotting Analysis

Expression of FCV proteins was verified via Immunoblotting. CRFK, F81, and Fc3Tg cells were infected with FCV HN/ZZ/2025 at a multiplicity of infection (MOI) of 0.05. At 24 h post-infection (hpi), cells were harvested and lysed using RIPA buffer (Beyotime, P0013B, Shanghai, China). Protein samples were resolved by SDS-PAGE and transferred onto polyvinylidene difluoride membranes (Millipore, Billerica, MA, USA). The membranes were blocked with 5% non-fat milk for 1 h at room temperature and subsequently incubated overnight at 4 °C with a primary rabbit anti-VP1 polyclonal antibody. After washing, the membranes were incubated with a corresponding HRP-conjugated secondary antibody for 1 h at room temperature. Protein bands were visualized using Luminata Crescendo Western HRP Substrate (Millipore, WBLUR0500) and captured with a GE AI600 imaging system (GE, Boston, MA, USA).

#### 2.7.3. Indirect Immunofluorescence Assay (IFA)

For IFA, CRFK, F81, and Fc3Tg cells were infected with FCV (MOI = 0.05) for 24 h. Cells were fixed with 4% paraformaldehyde for 10 min and permeabilized with 0.1% Triton X-100 in PBS for 10 min. Following blocking with PBS containing 10% FBS for 1 h, the cells were incubated with a primary polyclonal antibody targeting the major capsid protein (VP1) for 1 h at 37 °C. After three washes with PBS, the cells were incubated with an Alexa-Fluor-conjugated secondary antibody (diluted in 10% FBS) for 1 h at room temperature. Viral localization was visualized and photographed using an Olympus fluorescence microscope.

#### 2.7.4. Replication Kinetics and RT-qPCR Analysis

To evaluate FCV replication kinetics, CRFK, F81, and Fc3Tg cells in 35 mm dishes were infected with FCV at an MOI of 0.05. After 1 h of adsorption at 37 °C, the cells were washed three times with PBS to remove unbound virions. Cell samples were collected at 0, 6, 12, 18, 24, 30, 36, 48, and 60 hpi and subjected to total RNA extraction. Viral loads were quantified by real-time fluorescent quantitative PCR (RT-qPCR) targeting the VP1 gene. A standard curve was generated using 10-fold serial dilutions of a pET21b-FCV-VP1 recombinant standard plasmid. The RT-qPCR was performed in triplicate using the following primers: FCV VP1-Fw: 5′-TTGCAAAGAGCATGGGGTGA-3′, FCV VP1-Rv: 5′-CCAAGCATTGCCAGTGTGAC-3′. The viral copy numbers were calculated by mapping the mean cycle threshold (Ct) values against the established standard curve.

### 2.8. Transmission Electron Microscopy (TEM) and Physicochemical Characterization

#### 2.8.1. Morphological Characterization

To characterize the morphology of FCV virions, the virus was propagated in CRFK cells, and the resulting culture supernatant was harvested. The virus was concentrated via ultracentrifugation and subsequently purified using an iodixanol (OptiPrep) density gradient. The presence of purified FCV was confirmed by PCR and SDS-PAGE analysis. For TEM visualization, the purified viral suspension was applied to a carbon-coated copper grid (supporting membrane) and negatively stained with 1% phosphotungstic acid. Excess stain was removed using filter paper, and the grids were dried at room temperature. The virion architecture and diameter were examined using a transmission electron microscope (JEOL, JEM-1400, JEOL Ltd., Tokyo, Japan) at an accelerating voltage of 80–100 kV.

#### 2.8.2. Physicochemical Stability

The environmental stability of FCV HN/ZZ/2025 was assessed using cultures with a starting titer of 1 × 10^7.5^ TCID_50_/mL. The viral suspensions were subjected to various stressors, including thermal treatment (50 °C), acidic conditions (pH 3.0), alkaline conditions (pH 10.0), and exposure to organic solvents (chloroform and ether). Following treatment, the samples were neutralized or processed as required and inoculated onto confluent CRFK cell monolayers to monitor for CPE. Culture supernatants were harvested 48 h post-inoculation, and residual viral infectivity was quantified using the TCID_50_ assay.

### 2.9. Preparation of Rabbit Polyclonal Antibody Against VP1 Protein

The FCV VP1 gene was amplified and subcloned into the pET21b and pGEX-4T-1 prokaryotic expression vectors (Figure S1). Recombinant plasmids were transformed into Escherichia coli BL21 (DE3) competent cells. The transformed bacteria were cultured at 37 °C until the OD600 reached 0.6–0.8, at which point protein expression was induced with 0.5 mM isopropyl-β-D-thiogalactopyranoside for 12 h at 18 °C. The cells were harvested by centrifugation, resuspended in 30 mL of lysis buffer, and disrupted via sonication.

The expression of recombinant VP1 protein was verified by Western blot using anti-His-tag and anti-GST-tag antibodies. The His-tagged and GST-tagged VP1 proteins were purified using Ni-NTA Resin (GenScript, L00250, Nanjing, China) and Glutathione Resin (GenScript, L00206), respectively, according to the manufacturer’s protocols. The purified VP1 protein was used as an immunogen to immunize one 4-month-old New Zealand White rabbit. For each immunization, 100 μg of purified VP1 protein was emulsified with Freund’s adjuvant (Sigma, Burlington, MA, USA) and administered subcutaneously. Immunizations were performed at 14-day intervals for a total of three immunizations. A fourth booster immunization was subsequently administered with VP1 protein alone. Serum was collected after the final booster immunization and the antibody titre was approximately 1:50,000. The specificity and titer of the rabbit anti-VP1 serum were validated via Western blot and IFA for the detection of FCV (Figure S2).

### 2.10. Animal Infection Studies

Six two-month-old domestic cats, including four females and two males, were enrolled in the challenge study. The cats were common domestic cats, and were confirmed negative for FCV, FPV, and FHV by PCR before the experiment. Prechallenge serum samples were also collected for ELISA and virus neutralization assays. All six cats were negative for detectable antibodies against FCV, FPV, and FHV, and no neutralizing antibodies against these viruses were detected before challenge. The animals were housed in a dedicated animal facility at Henan Agricultural University with adequate ventilation and received routine husbandry, including free access to food and water, throughout the study. Following a 7-day acclimatization period, the cats were randomly assigned to two groups (n = 3/group). The challenge group was intranasally inoculated with 1 mL of MEM containing 1 × 10^7^ TCID_50_/0.1 mL of FCV HN/ZZ/2025, while the control group received an equal volume of virus-free MEM. Clinical health status—including pyrexia, weight fluctuations, mental status, appetite, and mucosal ulceration—was recorded daily using a standardized clinical scoring system. Oral and nasal swabs were collected daily for viral shedding analysis via RT-qPCR. On day 21 post-challenge, all cats were humanely euthanized for systemic pathological evaluation. Heart, liver, spleen, lung, kidney, and trachea tissues were harvested to quantify viral burden and evaluate histopathological alterations. Animal procedures were conducted in accordance with the approved animal welfare protocol and the principles of Replacement, Reduction, and Refinement (3Rs). Appropriate measures were taken throughout the experiment to minimize animal discomfort and distress. Animals were euthanized by intravenous administration of pentobarbital sodium (100 mg/kg), as described in Section 2.1.

A clinical scoring system was established to comprehensively assess the health status of the experimental cats. Eight parameters were evaluated: body temperature, body weight, mental status, appetite, ocular discharge, nasal discharge, oral ulceration, and survival status. Body temperature was scored as 0 when it ranged from 38.0 to 39.5 °C, 1 when it was ≥39.5 °C, and 2 when it was <37.0 °C. A body weight change of <3% was assigned a score of 0, whereas a weight loss of ≥3% was assigned a score of 2. Mental status was scored as 0 for normal behavior and 1 for lethargy. Appetite was scored as 0 for normal food intake, 1 for reduced appetite, and 2 for anorexia. Ocular discharge was scored as 0 when absent and 1 when present. Nasal discharge was scored as 0 when absent, 1 for mild discharge, and 2 for moderate to severe discharge. Oral ulceration was scored as 0 when absent, 1 when small and few lesions were present, and 2 when large and multiple lesions were present. Survival status was scored as 0 for surviving animals and 10 in the event of death.

### 2.11. Viral RNA Load Determination

The viral burden in harvested tissues, as well as oral and nasal swabs from infected cats, was quantified via absolute RT-qPCR following experimental challenge. Total viral RNA was extracted from 200 μL of swab supernatants or tissue homogenates using the TIANamp Virus DNA/RNA Kit (TIANGEN, DP315, Beijing, China) according to the manufacturer’s instructions. Subsequent cDNA synthesis was performed using HiScript IV All-in-One Ultra RT SuperMix (Vazyme, R433-01).

Quantification of FCV genomic copy numbers was conducted as previously described. Briefly, a 179 bp fragment of the FCV VP1 gene was cloned into the pET21b vector to serve as a standard. Absolute quantification was achieved using a standard curve generated from 10-fold serial dilutions of the recombinant plasmid (10^1^–10^12^ copies/μL). The RT-qPCR was performed in triplicate using ChamQ SYBR qPCR Master Mix (Vazyme, Q341-02) on a real-time PCR system. The primer sequences utilized were FCV VP1-Fw: 5′-TTGCAAAGAGCATGGGGTGA-3′, FCV VP1-Rv: 5′-CCAAGCATTGCCAGTGTGAC-3′.

### 2.12. Histopathological and Immunohistochemical (IHC) Analyses

#### 2.12.1. Histopathology

During necropsy, representative samples of the heart, liver, spleen, lung, kidney, and trachea were systematically collected. Tissues were fixed in 4% paraformaldehyde at room temperature for 72 h, followed by dehydration in a graded ethanol series, clearing in xylene, and embedding in paraffin wax. The paraffin-embedded blocks were sectioned at a thickness of 5 μm using a microtome (Servicebio, Wuhan, China). For histopathological evaluation, the sections were deparaffinized, rehydrated, and stained with hematoxylin and eosin. Pathological alterations were examined and photographed under a light microscope.

#### 2.12.2. IHC

For the in situ detection of FCV antigens, IHC was performed on tissue sections. Briefly, slides were deparaffinized, rehydrated, and subjected to heat-induced epitope retrieval using EDTA buffer (pH 9.0). Endogenous peroxidase activity was quenched, and sections were blocked with 5% BSA for 30 min at room temperature. The sections were then incubated with the primary rabbit anti-FCV VP1 pAb overnight at 4 °C.

For IHC, sections were subsequently incubated with an HRP-labeled anti-rabbit IgG secondary antibody (1:500, Servicebio). FCV VP1 antigen-positive cells were visualized with 3,3′-Diaminobenzidine, yielding a characteristic brown precipitate, and nuclei were counterstained with hematoxylin. The procedures mirrored those described in Section 2.7, with the additional inclusion of the EDTA antigen retrieval step. All slides were analyzed under a light or fluorescence microscope to evaluate the distribution and intensity of the viral antigen.

### 2.13. Statistical Analysis

Quantitative data were analyzed using GraphPad Prism 8.0 software. Statistical significance between groups was determined using Student’s *t*-test. Data are presented as mean ± SD, and *p*-values < 0.05 were considered statistically significant (* *p* < 0.05, ** *p* < 0.01, *** *p* < 0.001).

## 3. Results

### 3.1. FCV Sample Collection, Detection and Next-Generation Sequencing Analysis

**Figure 1:**
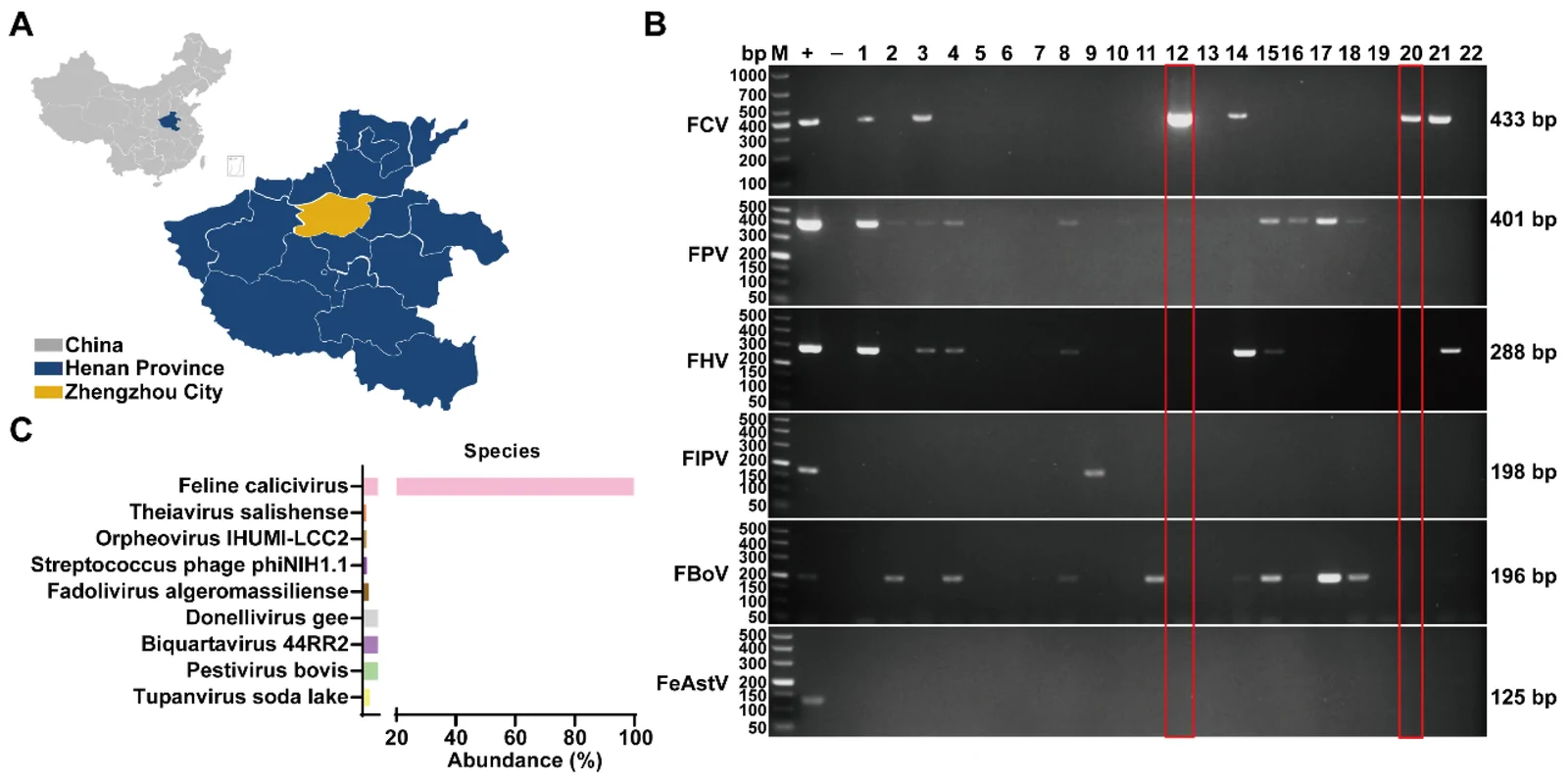
Sample Collection and Identification. (**A**) The sample collection site was labeled with a yellow pattern. The base layer of this modified map originated from National Earth System Science data center, National Science & Technology infrastructure of China (http://www.Geodata.cn) (**B**) PCR identification of FCV and other viruses (FPV, FHV, FIPV, FBoV, and FeAstV) in oral and nasal swabs. (**C**) NGS statistical results revealed FCV as the only identified virus in samples.

In January 2025, oral and nasal swabs were collected from 22 cats clinically suspected of FCV infection at a feline trading market in Zhengzhou, Henan Province, China. Oral and nasal swabs from each cat were combined as one clinical sample, resulting in 22 samples in total (Figure 1A). Total DNA/RNA was extracted, and subsequent PCR analysis confirmed FCV positivity in the specimens. Screening for other feline viruses revealed that the majority of cases involved co-infections, with only two of the 22 samples (2/22) identified as FCV-only infections. Aside from FCV, feline panleukopenia virus (FPV), feline bocavirus (FBoV), and feline herpesvirus (FHV) exhibited the highest prevalence rates (Figure 1B). To investigate potential cryptic pathogens, oral and nasal swabs were subjected to next-generation sequencing. Comprehensive analysis against the NCBI-nt and RVDB databases was performed to identify any associated microbial signatures. After filtering host-derived sequences and performing taxonomic annotation, FCV-specific sequences were found to be the most abundant viral reads, comprising nearly 100% of the viral population (Figure 1C). Consequently, FCV-positive samples from single-infection cases were selected for further virus isolation.

### 3.2. Isolation, Characterization, and Purification of FCV

**Figure 2:**
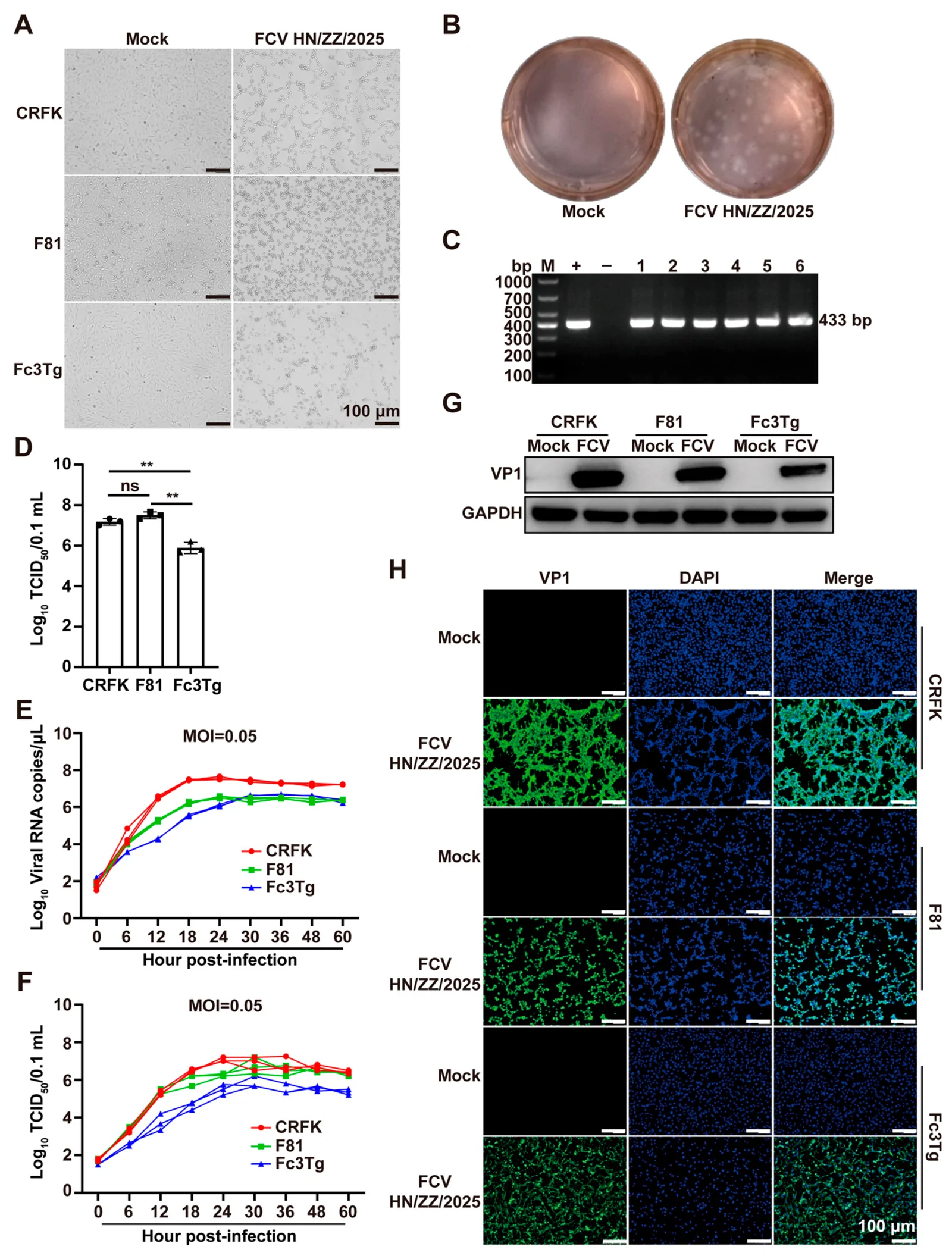
Isolation and characterization of FCV. (**A**) Virus-induced CPE in FCV-infected CRFK, F81 and Fc3Tg cells that are visible on light microscopy. (**B**,**C**) PCR detection of FCV in plaque-purified samples following serial passages. (**D**) Viral titers of FCV Strain HN/ZZ/2025 in CRFK, F81, and Fc3Tg cells determined by TCID_50_. Data are presented from three independent experiments (n = 3), with individual data points shown. (**E**,**F**) Growth curves of FCV in CRFK, F81, and Fc3Tg cells. Viral RNA copies at 0, 6, 12, 18, 24, 30, 36, 48 and 60 hpi determined by RT-qPCR and TCID_50_ assay. Data are presented from three independent experiments (n = 3), with individual data points shown. (**G**) Immunoblotting was used to detect the expression of viral protein VP1 in CRFK, F81, and Fc3Tg cells infected with FCV HN/ZZ/2025. (**H**) FCV-infected CRFK, F81, and Fc3Tg cells detected by IFA, the infected cells were stained with a rabbit serum raised against the FCV VP1 protein. Scale bar, 100 μm. ** *p* < 0.01.

CRFK cells were inoculated with supernatants from processed FCV-positive oral and nasal swab. Inoculated CRFK cells exhibited significant CPE—characterized by cell rounding, shrinkage, and a “grape-like” morphology—starting at 12 h post-infection (hpi), whereas control cells remained confluent (Figure 2A). Similar CPE was observed in F81 and Fc3Tg cells, though cytopathic changes in Fc3Tg cells were delayed, appearing only after 24 hpi. Successful isolation was followed by plaque purification, with distinct plaques visible after three successive rounds (Figure 2B). Stable FCV replication was confirmed through six consecutive passages in CRFK cells, with FCV-specific genomic fragments consistently detected in the culture supernatants via PCR (Figure 2C). The viral titers (TCID_50_) in CRFK, F81, and Fc3Tg cells were 10^7.18^, 10^7.50^, and 10^5.89^ per 0.1 mL, respectively (Figure 2D). Replication kinetics, assessed using an MOI of 0.05, demonstrated that the isolate reached peak viral loads at 24 hpi in CRFK and F81 cells, and at 36 hpi in Fc3Tg cells (Figure 2E,F). These results indicate that the virus reached its peak replication level earlier in CRFK and F81 cells than in Fc3Tg cells. To validate the isolation at the protein level, the VP1 protein (encoded by ORF2) was expressed to generate a rabbit-derived polyclonal antibody (pAb). IFA and immunoblotting using this anti-VP1 pAb confirmed robust viral replication and specific protein expression across all three cell lines (Figure 2G,H and Figure S2). This newly isolated FCV strain was designated strain HN/ZZ/2025 (GenBank accession number: PX408781).

### 3.3. Virus Particles and Physicochemical Properties of the FCV Strain HN/ZZ/2025

**Figure 3:**
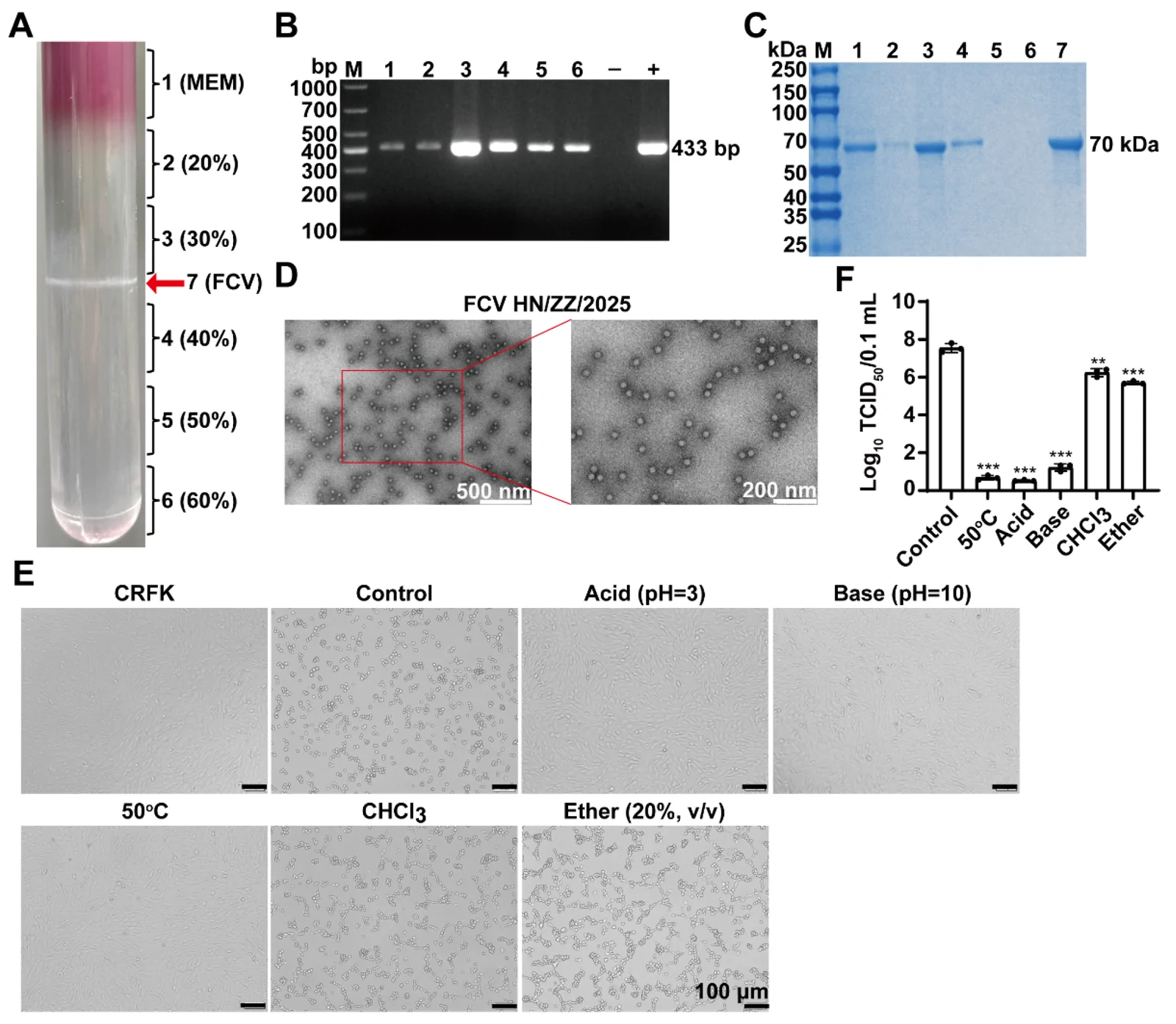
Virus particle morphology and Physicochemical properties of the FCV strain HN/ZZ/2025. (**A**) Purification of virus by density gradient centrifugation with iodixanol. (**B**,**C**) Determination of viral nucleic acid and protein band positions by PCR and SDS-PAGE. 1: Less than the 20% iodixanol, 2: 20% iodixanol, 3: 30% iodixanol, 4: 40% iodixanol, 5: 50% iodixanol, and 6: 60% iodixanol; 7: purified FCV preparation collected after density-gradient purification; “+” and “−” indicate the positive and negative controls, respectively. (**D**) Electron microscopic examination of morphology of FCV particles. Negatively stained virions purified from FCV-infected CRFK cells. Scale bar, 200 nm and 500 nm. (**E**,**F**) Identification of physicochemical properties of FCV strain HN/ZZ/2025. The CPE in CRFK cells infected with FCV treated at 50 °C or exposed to acidic, alkaline, chloroform, or ether conditions were observed under a microscope (**E**), and viral titers were determined by TCID_50_ (**F**). Scale bar, 100 μm. ** *p* < 0.01, *** *p* < 0.001.

The virus was purified using an iodixanol density gradient, with viral particles primarily partitioning into the 30% layer, as confirmed by PCR and SDS-PAGE (Figure 3A–C). Subsequent visualization via transmission electron microscopy (TEM) revealed typical icosahedral virions with diameters ranging from 35 to 40 nm (Figure 3D). The physicochemical stability of the HN/ZZ/2025 isolate was further characterized under various environmental stressors. Compared to the untreated control group, the isolate was completely inactivated after incubation at 50 °C for 30 min, and no residual infectivity was detected following treatment at pH 3.0 or pH 10.0. Conversely, the virus remained highly infectious and induced characteristic CPE after exposure to ether or chloroform. These findings indicate that strain HN/ZZ/2025 is thermolabile and sensitive to extreme pH conditions but remains resistant to lipid solvents, consistent with the properties of a non-enveloped virus (Figure 3E,F).

### 3.4. Genomic Characteristics and Phylogeny Analysis of FCV HN/ZZ/2025

**Figure 4:**
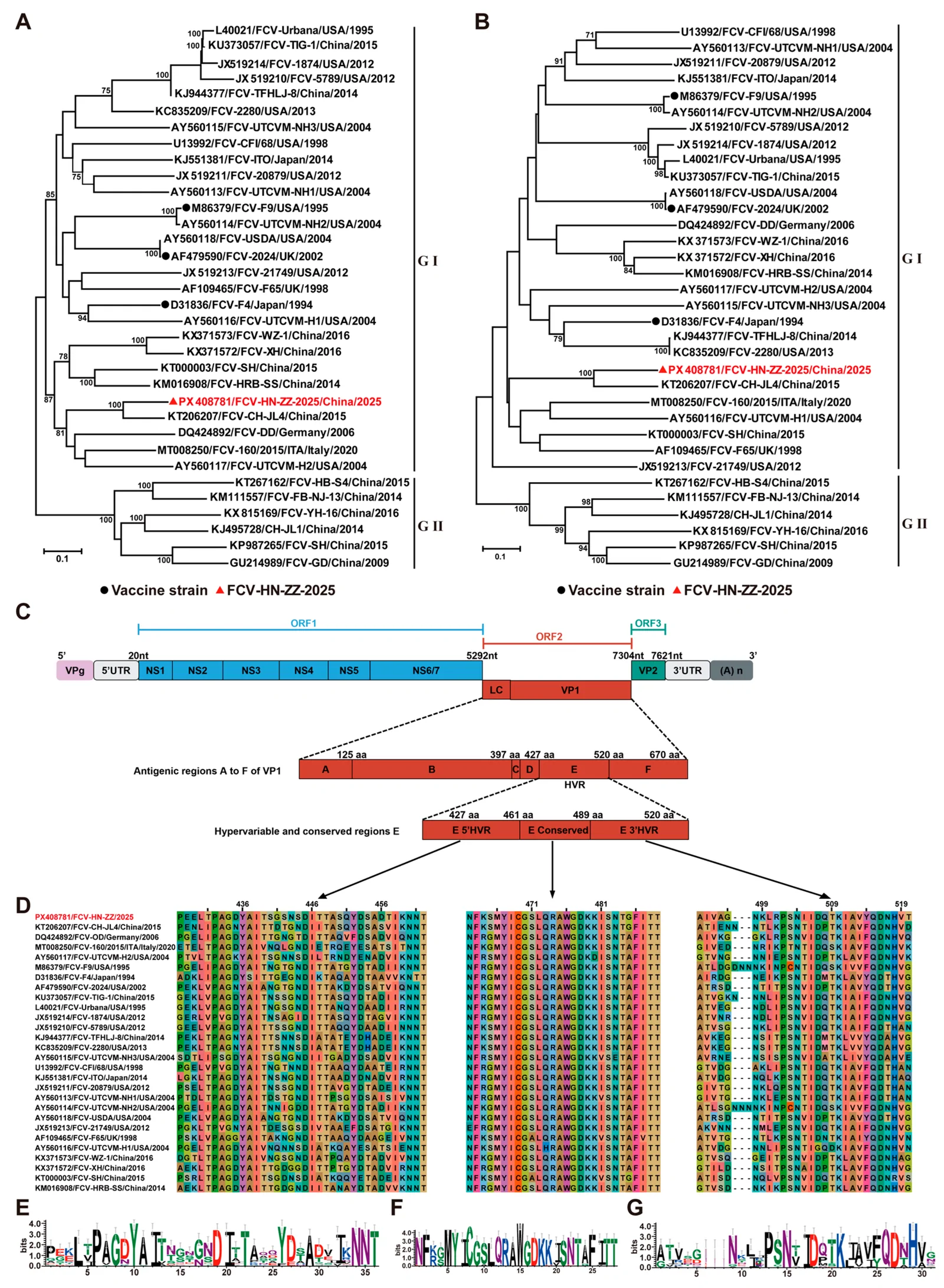
Phylogenetic analysis and amino acid sequence alignment of the isolates in this study. Phylogenetic analysis of isolates based on complete genome (**A**) and VP1 (**B**). The phylogenetic tree was generated using the maximum likelihood (ML) method implemented in the program MEGA 7.0. Bootstrap values are represented as a percentage based on 1000 replicates. (**C**) The genome organization of FCV HN/ZZ/2025 with open reading frames (ORF) 1–3, antigenic regions (**A**–**F**) of the VP1 capsid precursor protein, hypervariable and conserved regions of antigenic region E. VPg, virus protein, genome linked; LC, leader of capsid protein; HVR, hypervariable region. (**D**) Amino acid sequence alignment based on VP1. The abbreviation nt denotes nucleotides. (**E**–**G**) The conservation of amino acids in the VP1 E region, including the 5′ hypervariable region (**E**), the conserved region (**F**), and the 3′ hypervariable region (**G**), was analyzed using WebLogo.

The complete genome sequence of strain HN/ZZ/2025 was deposited in GenBank (accession number: PX408781). Phylogenetic trees were reconstructed based on the whole-genome and VP1 sequences of HN/ZZ/2025 in conjunction with 33 reference sequences (Figure 4A,B). Phylogenetic analysis identified the isolate as a genotype GI strain (classified by VP1 [38]), clustering closely with the Chinese FCV strain CH-JL4. The isolate shared its highest nucleotide and amino acid identities with CH-JL4 at 85.7% and 62.8%, respectively. Comparison with global FCV isolates revealed nucleotide identities ranging from 75.6% to 85.7% and amino acid identities from 46.3% to 62.8% (Table 1). Amino acid alignment localized the E region of the VP1 protein to residues 427–524. The 5′ (427–461 aa) and 3′ (490–520 aa) hypervariable regions exhibited low conservation, whereas the intervening sequence (462–489 aa) remained relatively conserved and likely constitutes the structural framework of the E domain. These genomic features confirm that HN/ZZ/2025 is a representative genotype GI variant (Figure 4C,D). The conservation of amino acids in the VP1 E region was analyzed using WebLogo (version v3.) (Figure 4E–G).

### 3.5. Clinical Signs of Cats Infected with the FCV HN/ZZ/2025 Strain

**Figure 5:**
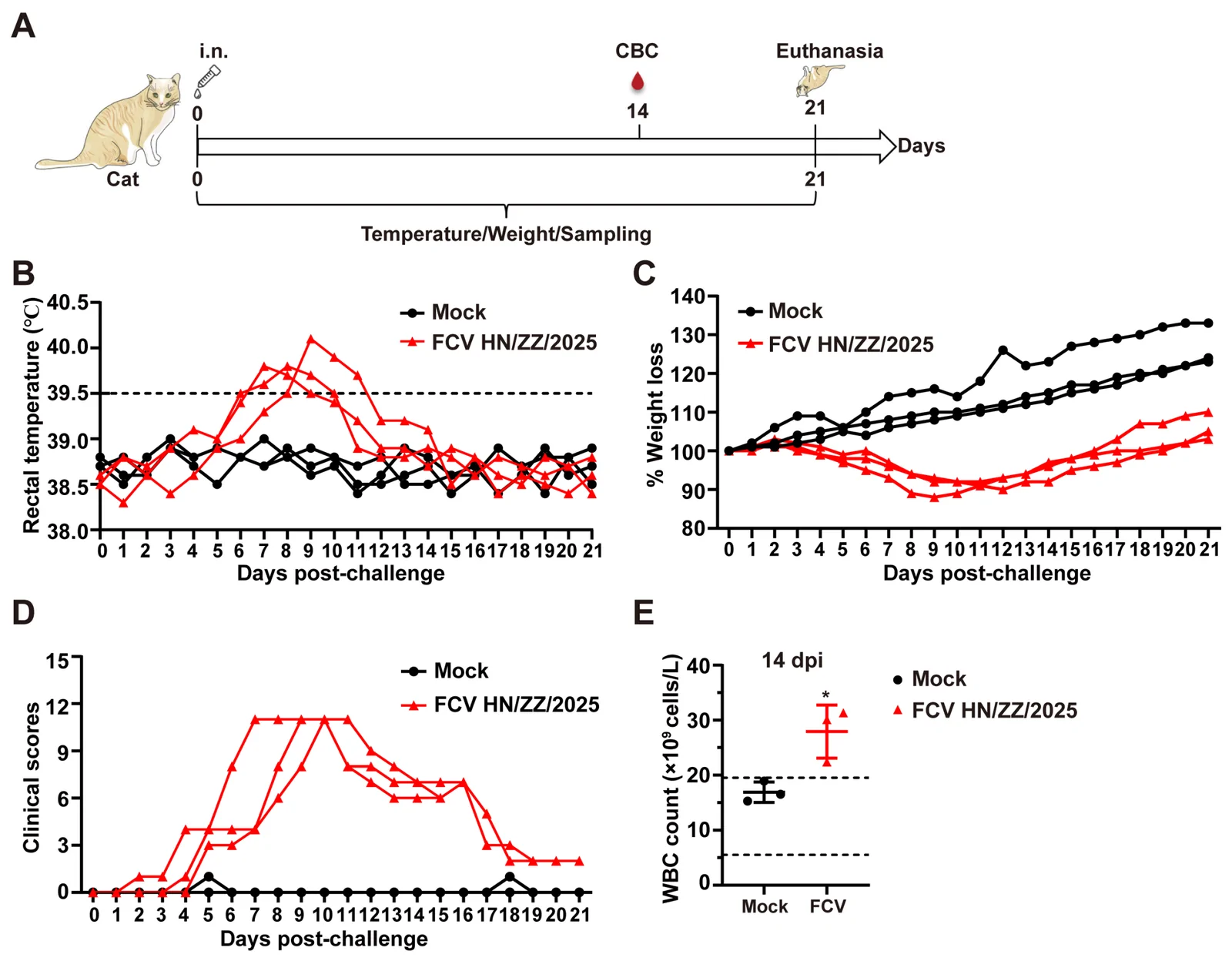
Clinical sign monitoring in cats infected with FCV HN/ZZ/2025. (**A**) Schematic diagram of the experimental infection and monitoring schedule following intranasal inoculation with FCV HN/ZZ/2025. (**B**) Changes in rectal temperature of cats following intranasal inoculation with FCV HN/ZZ/2025 or MEM (control). (**C**) Body weight changes in infected and control cats during the observation period. (**D**) Clinical score dynamics based on body temperature, body weight, mental status, appetite, ocular discharge, nasal discharge, oral ulcers, and survival status. (**E**) White blood cell (WBC) counts in infected and control cats at 14 dpi. * *p* < 0.05.

To evaluate pathogenicity, two-month-old cats were enrolled in a challenge study. The experimental group was intranasally inoculated with 1 × 10^7^ TCID_50_/0.1 mL of HN/ZZ/2025, while the negative control group received an equivalent volume of MEM. Clinical parameters, including body weight, rectal temperature, and symptomatic scores, were monitored daily; a complete blood count was performed at 14 days post-infection (dpi) (Figure 5A). In the infected group, pyrexia peaked at 39.8–0.2 °C by 9 dpi and remained significantly elevated relative to the control group throughout the observation period (Figure 5B). Infected cats exhibited significant weight loss peaking between 8 and 14 dpi, likely secondary to anorexia, dysphagia caused by oral ulceration, and systemic inflammation (Figure 5C). Clinical scores rose sharply after 3 dpi, peaking at 8–10 dpi before gradually declining (Figure 5D). Notably, significant leukocytosis was observed at 14 dpi (Figure 5E), which may primarily reflect a systemic inflammatory response associated with FCV infection. Although secondary bacterial factors cannot be completely excluded, no specific bacteriological investigations were performed in the present study, and therefore their contribution could not be determined.

### 3.6. Pathogenicity and Tissue Tropism of the FCV Strain HN/ZZ/2025 in Cats

**Figure 6:**
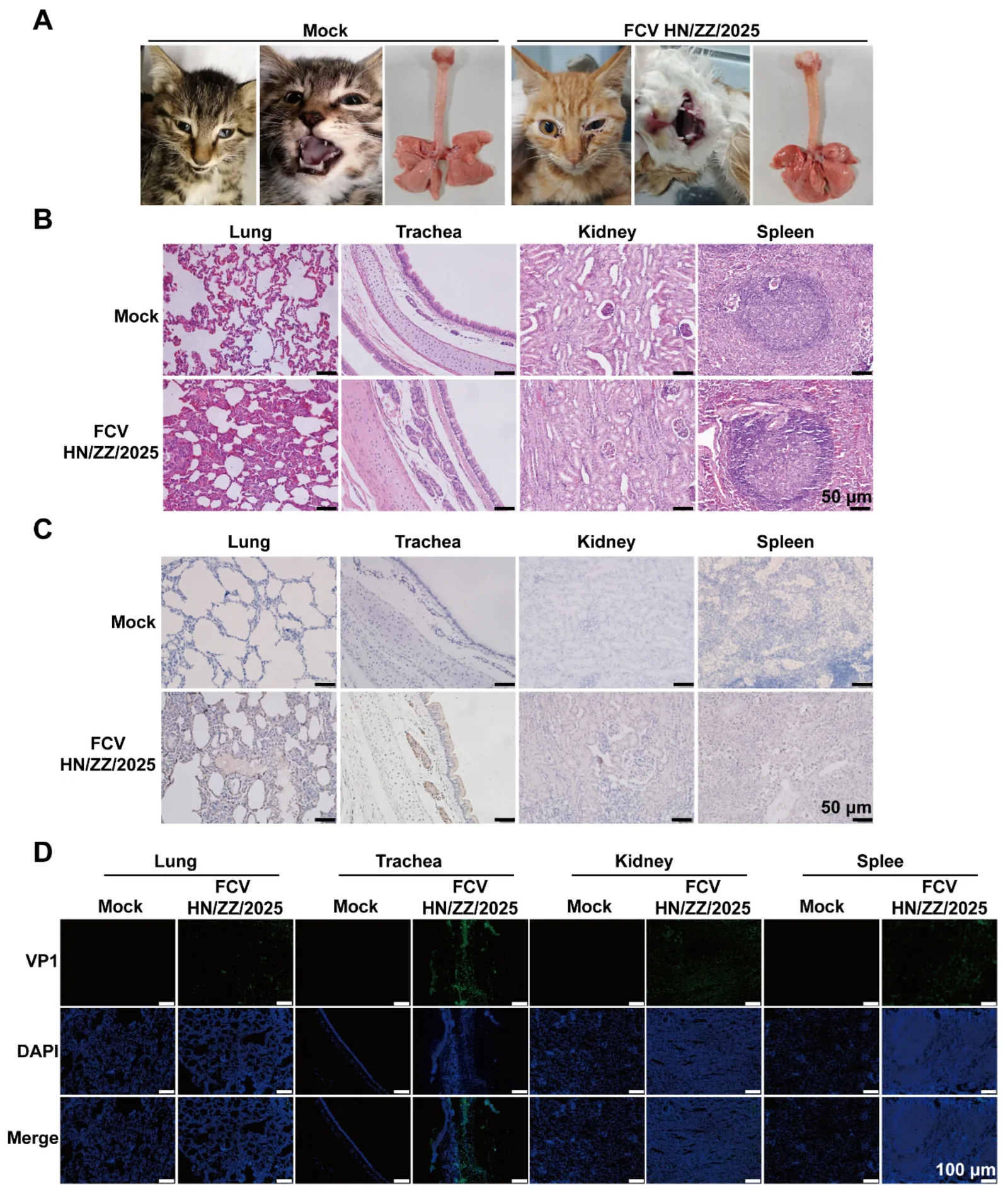
Pathological lesions and tissue tropism of FCV strain HN/ZZ/2025 in experimentally infected cats. (**A**) Clinical signs in cats, and macroscopic examination of lung lesions. (**B**–**D**) Necropsy examinations, histopathology, IHC and IFA of the lungs, trachea, kidneys, and spleen of the FCV challenge cats at 21 dpi. (**B**) Histopathological changes in tissues of FCV-infected cats revealed by hematoxylin and eosin (H&E) staining. (**C**) IHC and IFA detection FCV VP1 antigen-positive signals in the corresponding tissues of infected cats. Scale bar, 50 μm and 100 μm.

At 21 dpi, cats were euthanized for necropsy. Challenged cats presented with typical respiratory signs, including lacrimation, increased ocular discharge, and severe gingival congestion with associated ulceration. Necropsy revealed diffuse pulmonary hyperemia with multifocal petechial hemorrhages and mild tracheal submucosal edema in the infected group (Figure 6A). Histopathological analysis confirmed interstitial pneumonia, characterized by extensive inflammatory cell infiltration and pulmonary edema, alongside tracheal epithelial desquamation and mucosal congestion. Renal tubular epithelial cells showed degenerative changes, including vacuolization, while focal necrosis was identified in the spleen (Figure 6B). Following IHC and IFA, significant FCV-specific (VP1) signals and fluorescence were localized to the lungs, trachea, kidneys, and spleen (Figure 6C,D), whereas the heart and liver remained negative. These findings demonstrate that HN/ZZ/2025 induces systemic histopathological damage, with a primary tropism for respiratory and renal tissues.

### 3.7. Detection of Virus Shedding, Viral Load, and Protein Expression

**Figure 7:**
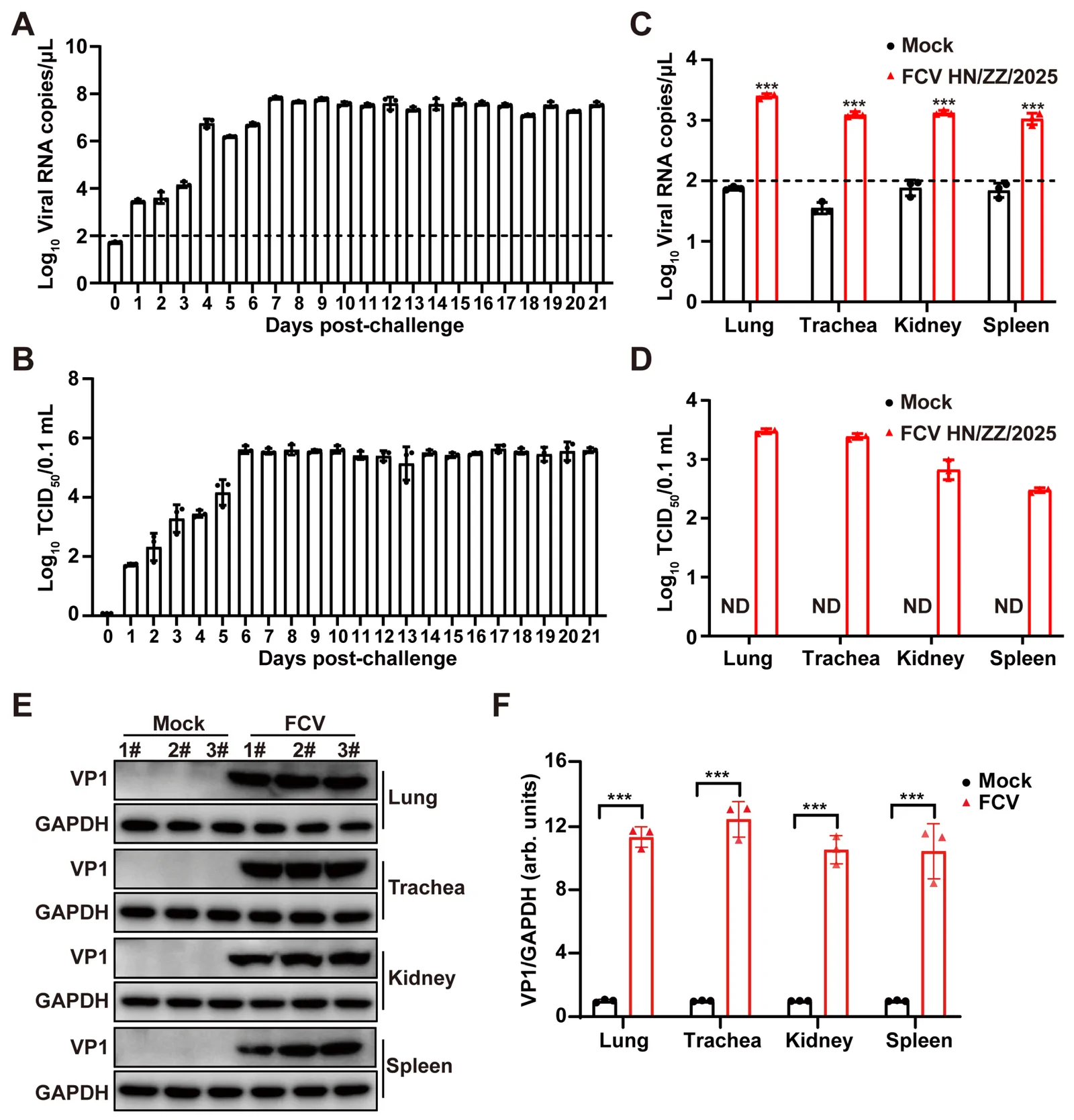
Viral shedding and tissue distribution of FCV HN/ZZ/2025 in infected cats. (**A**,**B**) Dynamic viral shedding in oral and nasal swabs collected from infected cats following intranasal inoculation, as determined by RT-qPCR and TCID_50_ assay. (**C**,**D**) Cats were euthanized at 21 dpi, and different organs were collected for determination of viral RNA loads by RT-qPCR (**C**) and infectious viral titers by TCID_50_ assay (**D**). (ND, not Detected). (**E**) Immunoblotting analysis showing the expression of FCV VP1 protein in tissues of infected cats. (**F**) Densitometric analysis of VP1 protein expression in tissues based on Image J, normalized to GAPDH. arb. units, arbitrary units. *** *p* < 0.001.

Viral shedding kinetics were evaluated by daily collection of oral and nasal swabs from infected cats and quantified by RT-qPCR and TCID_50_ assays. Viral RNA was first detected at 1 dpi, with viral RNA loads peaking between 5 and 7 dpi and remaining detectable until the end of the observation period at 21 dpi (Figure 7A). Infectious virus was also detected in oral and nasal swabs during the shedding period, with a similar trend to viral RNA levels (Figure 7B). At 21 dpi, viral RNA was detected in multiple organs of infected cats, whereas no viral RNA was detected in the corresponding tissues of control cats (Figure 7C). Consistent with the RT-qPCR results, infectious virus was detected in several tissues of infected cats by TCID_50_ assay (Figure 7D). These findings indicate systemic viral distribution, with the respiratory tract showing prominent viral detection. Immunoblotting further confirmed FCV VP1 protein expression within the infected tissues (Figure 7E). Collectively, these findings demonstrate that HN/ZZ/2025 is capable of efficient replication, persistent viral shedding, and systemic dissemination in experimentally infected cats, while no mortality was observed during the 21-day observation period.

## 4. Discussion

Driven by socio-economic development, the growing demand for psychological companionship has led to a rapid expansion of the companion animal market. It is estimated that over 600 million cats and 900 million dogs live alongside humans worldwide, with feline ownership being particularly prominent [1]. The “One Health” framework emphasizes the profound interconnectivity between human health, companion animals, and their shared environments. Research on feline viral infections not only enhances veterinary care but also informs the development of diagnostic tools and vaccines for related human pathogens.

In the present study, we characterized a genotype GI FCV strain, designated HN/ZZ/2025, newly isolated from a pet market in Zhengzhou, China. Phylogenetic analysis revealed that HN/ZZ/2025 is closely related to the Jilin strain CH-JL4. Notably, compared with the severe clinical manifestations and high mortality reported for some VS-FCV strains, cats challenged with HN/ZZ/2025 did not develop fatal disease during the 21-day observation period. However, the infected cats developed pyrexia, weight loss, oral ulceration, persistent viral shedding, and histopathological lesions, indicating that HN/ZZ/2025 can cause clinically and pathologically evident disease under the experimental conditions used in this study. High-density settings, such as shelters and pet markets–characterized by a frequent influx of animals with undocumented immunological histories—act as catalysts for FCV transmission and genetic diversification [39]. Phylogenetic clustering indicates genetic relatedness between HN/ZZ/2025 and FCV isolates reported from different geographic regions. However, the present analysis does not provide sufficient evidence to infer the direction or historical route of geographic dissemination.

FCV and FHV-1 remain the predominant viral agents of feline URTDs [5,6,7]. FCV is particularly notable for its high contagiousness and environmental stability. Our investigation into the physicochemical properties of HN/ZZ/2025 demonstrated that the isolate is thermolabile at 50 °C and sensitive to extreme pH (3.0 and 10.0). However, as a non-enveloped virus, its lack of a lipid envelope confers robust resilience against organic solvents like chloroform and ether. These findings suggest that effective biosecurity measures must prioritize disinfectants validated against non-enveloped viruses, such as sodium hypochlorite, potassium persulfate, or high-level heat treatment (≥60 °C) [40,41].

The ~7500 nt FCV genome is subject to high mutation rates due to the absence of proofreading activity during replication [42]. The VP1 protein, encoded by ORF2, is the primary structural protein and immunodominant target for neutralizing antibodies. We observed that while regions A, B, D, and F are conserved, the hypervariable E region (residues 427–524) exhibits significant diversity, which likely facilitates immune evasion and reduces vaccine efficacy. This region mediates interaction with the feline junctional adhesion molecule A (fJAM-A) receptor; its disruption is linked to the characteristic formation of oral ulcers. While a point mutation was identified in the ITTANQY neutralizing epitope of HN/ZZ/2025 [43], the PAGDY epitope remained relatively conserved, suggesting its potential utility as a target for broadly cross-reactive diagnostic assays [44].

Historically, FCV manifested as a low-pathogenicity pathogen until the emergence of VS-FCV variants characterized by high mortality (33–100%) [45]. Outbreaks of VS-FCV have been documented globally, spreading from the United States and Europe to Asia. In China, VS-FCV was first reported in 2016 in a Amur tiger [35] and has since been detected in domestic populations. Our experimental challenge demonstrated that HN/ZZ/2025 caused clinical disease and systemic tissue involvement but did not result in mortality during the observation period. The infectious dose used in the present study (1 × 10^7^ TCID_50_/0.1 mL per cat) was selected to establish a reproducible experimental infection model. This dose is comparable to that used in a previous FCV pathogenicity study, in which cats were challenged with 1 × 10^7^ TCID_50_/0.1 mL of FCV strains TIG-1, 2280, and F9 [35]. However, the use of a controlled high-dose inoculum may not fully reproduce the infection dynamics associated with natural FCV exposure. Therefore, the clinical manifestations, viral shedding, and tissue distribution observed in the present study should be interpreted in the context of the experimental challenge model. Although the observed disease severity appeared less pronounced than that reported for some VS-FCV strains such as 2280 and TIG-1, the systemic dissemination observed in the present study, together with typical respiratory involvement, highlights the broad tissue distribution of HN/ZZ/2025. However, genomic analysis did not reveal specific amino acid signatures consistently associated with hyper-virulence, suggesting that pathogenicity is likely polygenic and warrants further functional genomic investigation.

The persistent circulation of FCV within domestic cat populations serves as a valuable natural model for viral evolution. Effective management necessitates diagnostic synergy between clinical observation and laboratory confirmation via RT-qPCR, virus isolation, and immunohistochemistry [16]. Although RT-qPCR offers high sensitivity, the genetic heterogeneity of FCV requires cautious interpretation of results. Currently, clinical management remains largely supportive, as definitive antiviral therapies are lacking. While vaccination remains the cornerstone of prevention, the substantial antigenic variation observed in strains like HN/ZZ/2025 underscores the need for continuous vaccine optimization. In conclusion, effective FCV control requires an integrated approach combining early diagnosis, rigorous disinfection, and the development of vaccines with broader cross-protective efficacy.

This study has several limitations. First, the animal experiment included only three cats per group, which limited the statistical power and generalizability of the pathogenicity assessment. Second, no reference FCV strain with a well-characterized virulence phenotype was included for direct in vivo comparison; therefore, the relative pathogenicity of HN/ZZ/2025 should be interpreted with caution. Third, neutralizing antibody responses and seroconversion were not evaluated, and thus the host immune response following infection could not be characterized. Finally, the present study did not evaluate the immunogenicity, protective efficacy, safety, or genetic stability of HN/ZZ/2025. Therefore, further studies are required to determine the biological and immunological significance of this isolate and its potential applications in future FCV research.

## 5. Conclusions

In conclusion, this study describes the successful isolation and comprehensive characterization of FCV strain HN/ZZ/2025, a member of the dominant GI genotype. The isolate features distinct genomic variations within the immunodominant hypervariable E region and exhibits robust replication kinetics and broad cellular tropism across CRFK, F81, and Fc3Tg cell lines. While experimental infection caused clinical disease, systemic viral dissemination, and persistent viral shedding, no mortality was observed during the 21-day observation period. These findings characterize HN/ZZ/2025 as a newly isolated FCV strain with distinct molecular and biological characteristics and provide a useful isolate for future studies of FCV genetic diversity and pathogenesis.

## Figures and Tables

**Table 1:** The sequence identity within the FCV polyprotein at the nucleotide (upper right) and amino acid (lower left, boldface) levels calculated using the ClustalW method implemented in MegAlign. The compared strains were indicated as accession, strain name number and country. GenBank accession number: 1 (PX408781/FCV-HN-ZZ-2025); 2 (U13992/FCV-CFV/68/USA/1998); 3 (MT008250/FCV-160/2015/ITA/Italy/2020); 4 (M86379/FCV-F9/USA/1995); 5 (L40021/FCV-Urbana/USA/1995); 6 (KX371573/FCV-WZ-1/China/2016); 7 (KX371572/FCV-XH/China/2016); 8 (KU373057/FCV-TIG-1/China/2015); 9 (KT206207/FCV-CH-JL4/China/2015); 10 (KT000003/FCV-SH/China/2015); 11 (KM016908/FCV-HRB-SS/China/2014); 12 (KJ944377/FCV-TFHLJ-8/China/2014); 13 (KJ551381/FCV-ITO/Japan/2014); 14 (KC835209/FCV-2280/USA/2013); 15 (JX519214/FCV-1874/USA/2012); 16 (JX519213/FCV-21749/USA/2012); 17 (JX519211/FCV-20879/USA/2012); 18 (JX519210/FCV-5789/USA/2012); 19 (DQ424892/FCV-DD/Germany/2006); 20 (D31836/FCV-F4/Japan/1994); 21 (AY560118/FCV-USDA/USA/2004); 22 (AY560117/FCV-UTCVM-H2/USA/2004); 23 (AY560116/FCV-UTCVM-H1/USA/2004); 24 (AY560115/FCV-UTCVM-NH3/USA/2004); 25 (AY560114/FCV-UTCVM-NH2/USA/2004); 26 (AY560113/FCV-UTCVM-NH1/USA/2004); 27 (AF479590/FCV-2024/UK/2002); 28 (AF109465/FCV-F65/UK/1998); 29 (KT267162/FCV-HB-S4/China/2015); 30 (KM111557/FCV-FB-NJ-13/China/2014); 31 (KX815169/FCV-YH-16/China/2016); 32 (KJ495728/CH-JL1/China/2014); 33 (GU214989/FCV-GD/China/2009); 34 (KP987265/FCV-SH/China/2015).

Strain	1	2	3	4	5	6	7	8	9	10	11	12	13	14	15	16	17	18	19	20	21	22	23	24	25	26	27	28	29	30	31	32	33	34
1		78.2	80.4	77.7	78.1	78	78.3	78.2	85.7	80	80	78.4	77.5	79.3	78.2	77.8	77.5	78.1	79.3	78.4	79.1	78.9	78.4	78.5	77.8	78.3	79.1	78.3	76.3	76.6	76.2	76	75.6	76
2	48.6		79.4	79.3	80	79.1	78.5	79.9	78.8	79	79.2	79.6	79.7	79.8	80.1	79.3	80.3	80.1	78.9	80	79.8	79.6	79.3	79.3	79.3	80.9	79.7	80	76	77.3	77.3	76.9	76.7	76.2
3	53.3	52.4		78.7	79.4	79.4	79	79.3	81.3	80.3	80.5	79.3	78.6	80.4	79.3	78.6	79.1	79	80	78.9	79.5	81.5	79.3	79.4	78.7	79.8	79.5	79.3	75.7	76.4	76.6	76.4	76.1	76.3
4	48.2	49.6	49.8		79.7	78.5	78.7	79.7	78.5	78.2	78.6	79.6	78.7	79.8	80	79.5	79.1	79.5	78.6	80.3	80.1	78.5	78.8	78.9	98.3	79.8	80.1	79.3	76	76.6	76.5	76.8	75.7	76
5	49.4	50.5	51.8	50.1		79.3	79	97.2	79.2	79.4	80	91.9	79.1	80.1	94.7	79.6	79.8	91.7	79.3	80.1	80.3	79.1	79.1	80	79.8	79.8	80.3	79.6	76.2	77	77.1	77	76.7	76.7
6	48.8	50.1	51.5	49.3	49.8		88.1	79.4	78.2	79.3	82	79	77.5	79.1	79.3	78.7	78.4	79.1	78.6	78.9	79.1	78.4	78.2	79	78.5	78.8	79.1	78	75.8	76	76.5	76.7	76.1	75.8
7	49.5	49.7	50.6	48.9	49.5	66.8		79.1	78.3	78.9	81.9	79	77.4	79.5	79.6	78.7	78.9	78.5	78.6	78.7	79.1	78.8	78.2	78.9	78.9	78.4	79.1	78.3	75.3	76	75.9	76.5	76	75.5
8	49.9	50.1	51.6	50.3	85.7	50	49.6		79.1	79.5	80.1	93.6	79.1	80.1	96.1	79.8	79.8	92.6	79.3	80.4	80.5	79.3	79	80.1	79.8	79.9	80.5	79.7	76.4	77.2	77.3	77	76.7	76.8
9	62.8	49.4	54.4	48.2	50.6	48.4	48.3	50.7		80.6	80.5	79	77.8	79.6	79.2	78.9	78	78.6	79.6	78.8	79.4	79.8	78.6	79.2	78.5	79.3	79.3	79.6	76.2	77.1	76.7	77.1	76.4	76.5
10	52.5	49.2	54.4	47.8	50.2	50.4	49.9	50.6	53.4		84	79.5	78.5	80.7	80	79.4	79.7	79.4	80	79.9	79.8	79.4	80.2	80	78.2	79.9	79.7	79.8	76.3	77.5	76.9	77.4	76.3	76.3
11	53.4	50.3	53.5	49.3	51.5	55.4	55.6	51.7	53.2	58.7		79.5	78.8	79.3	80.1	79	79.1	79.8	79.2	79	79.6	79.8	78.4	79	78.6	79.4	79.6	79.2	77.3	76.9	76.2	76.6	76.5	76.4
12	49.7	50	51.8	50.2	74.9	49.6	49.4	78.6	50.2	51.1	50.9		78.9	86.3	91.2	79.6	79.8	88.8	79.3	80.6	80.4	79.4	79.7	80.4	79.6	79.6	80.4	79.8	75.9	76.9	76.8	77.3	76.3	76.6
13	49.2	51.2	50.7	50.1	50.7	48.2	48.3	51	48.7	49.2	50.9	50.4		79.5	79.5	79.2	80.6	79.6	78.3	79.8	79.6	78.3	79.3	79.5	78.8	80.6	79.6	78.4	75.8	76.2	76.3	75.9	76	75.3
14	51.4	51	53.5	50.3	51.3	50.1	50.3	51.2	50.8	52.6	50.4	64.2	51.4		80.1	79.7	81.2	80	79.1	81.1	80.6	79.4	80.1	81.3	79.9	80.8	80.6	80	76.2	77.2	77.1	77.4	76.5	76.3
15	49.5	50.4	51.8	50.9	80.1	49.5	50.5	83.4	50.5	51.1	52.1	73.3	51.7	51.3		80.1	80.3	91.4	79.3	80.6	80.6	79.4	79.3	80.2	80	80.2	80.6	80	76.2	77.2	77.4	77.4	76.9	76.9
16	48.3	49.5	49.8	49.3	50	48.5	49.3	50.5	49.1	50.4	49.7	50.4	50.5	50.5	51.3		79.4	79.4	78.8	80.4	80.1	78.7	79.8	79.1	79.5	79.7	80.1	79.8	76.4	76.8	77	76.8	76.2	76
17	48.1	51.8	51	48.9	50.4	48.9	49.6	51	48	51.1	49.8	51.3	52.6	53.5	51.8	49.8		80	78.8	80.4	80.1	79.1	79.1	80.4	79.1	81.9	80.1	79.4	76.4	76.3	77	76.6	76.8	76
18	49.6	50.5	51.2	50.1	73.7	49.2	48.5	75.9	49.7	50.4	51.1	68.6	51.4	51.1	73.2	49.8	51.1		78.9	80.3	80.3	79.4	79.4	79.7	79.7	80.2	80.2	79.5	76.2	77.3	77.6	77.1	76.7	76.5
19	50.8	49.5	52.9	47.7	49.8	49.1	49.7	50.1	50.9	51.8	50.7	50.7	49.3	50.7	50.1	49.1	49.5	49.3		79	79.5	80.9	79.1	78.4	78.6	79.2	79.5	78.6	75.7	76.7	76.3	76.1	75.6	76.2
20	49.3	51.2	51.1	51.2	51	49.8	49.5	51.9	48.8	51.1	50.5	52	52.7	52.9	52.2	51.8	52.3	52	49.6		80.9	79.5	81.2	79.6	80.2	80.1	80.9	80.3	76.4	77.6	77.3	77.7	77.1	77.1
21	51.5	50.5	52.2	50.4	51.7	50	49.9	52	50.1	51.5	51.5	51.9	52.5	52.9	52.6	50.8	51.4	51.9	50.4	52.7		80	80.1	79.8	80.1	80.3	99.9	79.7	76.6	77.3	78	78.2	77.4	76.8
22	50.4	50.4	54.7	47.4	48.8	48.8	49.5	49.7	51.6	50.7	51.9	49.7	49.2	50.7	49.6	48.3	49.4	49.1	52.6	50.7	50.7		78.5	79.5	78.5	80	80	79.5	77	77.3	77	77.1	76.6	76.6
23	50	49.6	51.3	48.6	49.6	49.1	48.8	49.1	49.6	51.6	49.4	50.5	51.8	52.3	50.4	51	49.6	50.1	50.5	54.6	52.1	48.7		78.6	78.7	79.7	80	79.7	76.1	76.8	76.7	76.7	76.7	76.6
24	49.3	49.9	51.3	49.3	51.4	48.7	48.7	51.3	49.7	51	49.6	51.8	51.5	53.5	51.2	49.2	51.6	50.4	47.8	50.6	50.6	50.6	49.9		79	80.7	79.7	78.9	76.5	76.8	76.8	77	76.1	75.4
25	48.5	49.7	49.8	88.5	50.4	49.6	49.8	50.5	48.8	48	49.6	50.3	50.2	50.7	51.3	49.4	49.1	50.5	48.1	50.9	50.6	47.2	48.7	49.6		79.7	80	79.4	76.1	76.7	76.5	76.7	75.9	76
26	49.1	52.6	52.5	50.2	50.2	48.7	48.5	50.7	50.6	50.7	50.9	50.1	53	51.8	51.2	50.1	54.5	50.5	49.7	51.3	50.9	50.9	51.5	51.9	50.2		80.3	80.1	76.1	76.9	77.1	77.1	76.6	76.5
27	51.4	50.4	52.2	50.4	51.6	49.8	49.7	52	50	51.5	51.4	51.8	52.3	52.8	52.5	50.6	51.2	51.8	50.3	52.6	92.1	50.5	51.9	50.5	50.5	50.8		79.7	76.6	77.2	77.9	78.1	77.3	76.7
28	49.2	50.6	50.5	49.8	50.1	47.6	47.6	50.4	50.7	49.7	49.8	50.9	49.3	50.9	51.2	50.1	49.8	50.2	48.4	51.7	50	49.4	50.3	48	49.5	51.1	50		75.7	76.6	76.6	76.8	76.1	76.5
29	47	45.6	46.9	45.6	45.7	46.1	45.4	46	46.4	46.4	49	45.5	46.8	46.3	45.7	46.1	46.6	44.8	44.9	46	47.6	47.6	45.5	46.5	45.9	45.4	47.6	44.6		82.7	78.9	79.8	78.9	79.5
30	47	46.8	47.4	45.8	46.3	45.9	46.1	46.8	47.2	47	47.6	46.5	45.9	46.5	46.8	45.5	45.6	47.1	46.5	47.3	46.6	47.2	45.8	45.4	46.1	45.5	46.4	45.1	55.3		80.1	81.3	79.9	80.3
31	46.6	47.4	47.2	45.3	46.3	46.6	44.9	46.5	45.6	46.6	46	46	47.3	46.8	46.6	46.4	46.5	47.5	45.4	46.5	47.2	46.4	46.1	46.2	45.4	46.9	47.1	45.5	49.6	50.5		82.9	79.9	80
32	46.3	46.7	47.7	46.1	46.6	46.7	46.3	46.7	47.4	47.8	46.3	47.3	46	47.1	47.4	46.7	46.2	46.8	46	48	49.1	47.4	46.5	47	46	46.7	48.9	45.8	51.4	52.7	56.3		80.2	80.4
33	46.6	47.1	46.6	44.3	46.4	46.2	46.2	46.7	46.5	46.7	47.6	45.9	46.4	46.7	46.9	45.8	46.4	46.7	45.8	47.9	48.4	46.7	47	45.3	44.9	45.8	48.2	44.9	51.1	51.6	51.9	53		84.5
34	46.8	45.2	46.7	45.3	46.1	45.2	44.3	46.5	46.7	45.8	47.4	46	45.8	45.8	46.5	45.1	44.9	45.6	46.2	46.8	46.1	45.8	46.7	43.9	45.5	46.8	46	45.8	50.9	51.6	50.9	52.4	60.6	

## Data Availability

The original contributions presented in the study are included in the article/Supplementary Material, further inquiries can be directed to the corresponding authors.

## Supplementary Materials

The following supporting information can be downloaded at: https://www.mdpi.com/article/10.3390/microorganisms14091958/s1, Supplementary Table S1: Identification primer sequences; Supplementary Table S2: Primer Sequences for FCV genome amplification; Supplementary Figure S1: Construction of pET21b-NT11-FCV VP1 and pGEX-4T-1-FCV VP1 expression vectors and prokaryotic expression of FCV VP1 protein; Supplementary Figure S2: Preparation and characterization of a rabbit-derived pAb to FCV VP1 protein.
