# Supplementary material for: Isolation, Molecular Characterization, and Pathogenicity Evaluation of a Genotype GI Feline Calicivirus Isolate
Source: Microorganisms. 2026 Sep 4;14(9):1958. doi: 10.3390/microorganisms14091958 (PMC13608943; doi:10.3390/microorganisms14091958)

Isolation, Molecular Characterization, and Pathogenicity Evaluation of a Genotype GI Feline Calicivirus Isolate

Jia-You Xing [1,2,3,†], Wen-Jie Xu [1,2,3,†], Zi-Xuan Fu [1,2,3], Ying-Na Xu [1,2,3], Jing-Yang Li [1,2,3], Jiang Wang [1,2,3,4], Bei-Bei Chu [1,2,3,4,5,6], Lei Zeng [1,2,3,]* and Sheng-Li Ming [1,2,3,]*

Supplementary Figure S1. Construction of pET21b-NT11-FCV VP1 and pGEX-4T-1-FCV VP1 expression vectors and prokaryotic expression of FCV VP1 protein. (A) PCR amplification of the FCV VP1 gene and double digestion of plasmid linearization. Lane assignments Lane M, DNA marker, Lane 1, amplified NT11-VP1 gene; Lane 2, Double digestion of plasmid pET-21b with *HindIII* and *XhoI* for vector linearization; Lane 3, amplified VP1 gene; Lane 4, Double digestion of plasmid pGEX-4T-1 with *XhoI* and *EcoRI* for vector linearization. (B) SDS-PAGE analysis of recombinant VP1 protein expression and purification. Lane assignments: Lane M, protein marker, Lane 1, uninduced E. coli BL21 (DE3) culture; Lane 2, IPTG induced E. coli BL21 (DE3) culture; Lane 3, supernatant of sonicated bacterial lysate; Lane4, pellet of sonicated bacterial lysate; Lane 5, Urea-denatured supernatant obtained during protein purification; Lane 6, Refolded supernatant obtained during protein purification; Lane 7, flow-through after binding of the supernatant to Ni-NTA resin; Lanes 8-14, fractions of the primary purified VP1 protein eluted with elution buffer containing 0, 10, 25, 50, 100, 250, and 500 mM imidazole, respectively. (C) SDS-PAGE analysis of recombinant GST-VP1 protein expression and purification. Lane assignments: Lane M, protein marker, Lane 1, uninduced E. coli BL21 (DE3) culture; Lane 2, IPTG induced E. coli BL21 (DE3) culture; Lane 3, supernatant of sonicated bacterial lysate; Lane4, pellet of sonicated bacterial lysate; Lane 5, flow-through after binding of the supernatant to Ni-NTA resin; Lane 6, Wash fraction containing nonspecifically bound proteins; Lane 7, Elution fractions of purified VP1 protein. (D) and (E) SDS-PAGE and immunoblotting verification of the recombinant VP1 protein Lane assignments: Lane M, protein marker, Lane 1, induced E. col B.21 (DE3 transformed with empty pET21b-NT11 and pGEX-4T-1 vector (negative control); Lane 2, induced E. coli BL21 (DE3) transformed with pET21b-NT11-FCV VP1 and pGEX-4T-1-FCV VP1; Lane 3, purified recombinant VP1 protein.

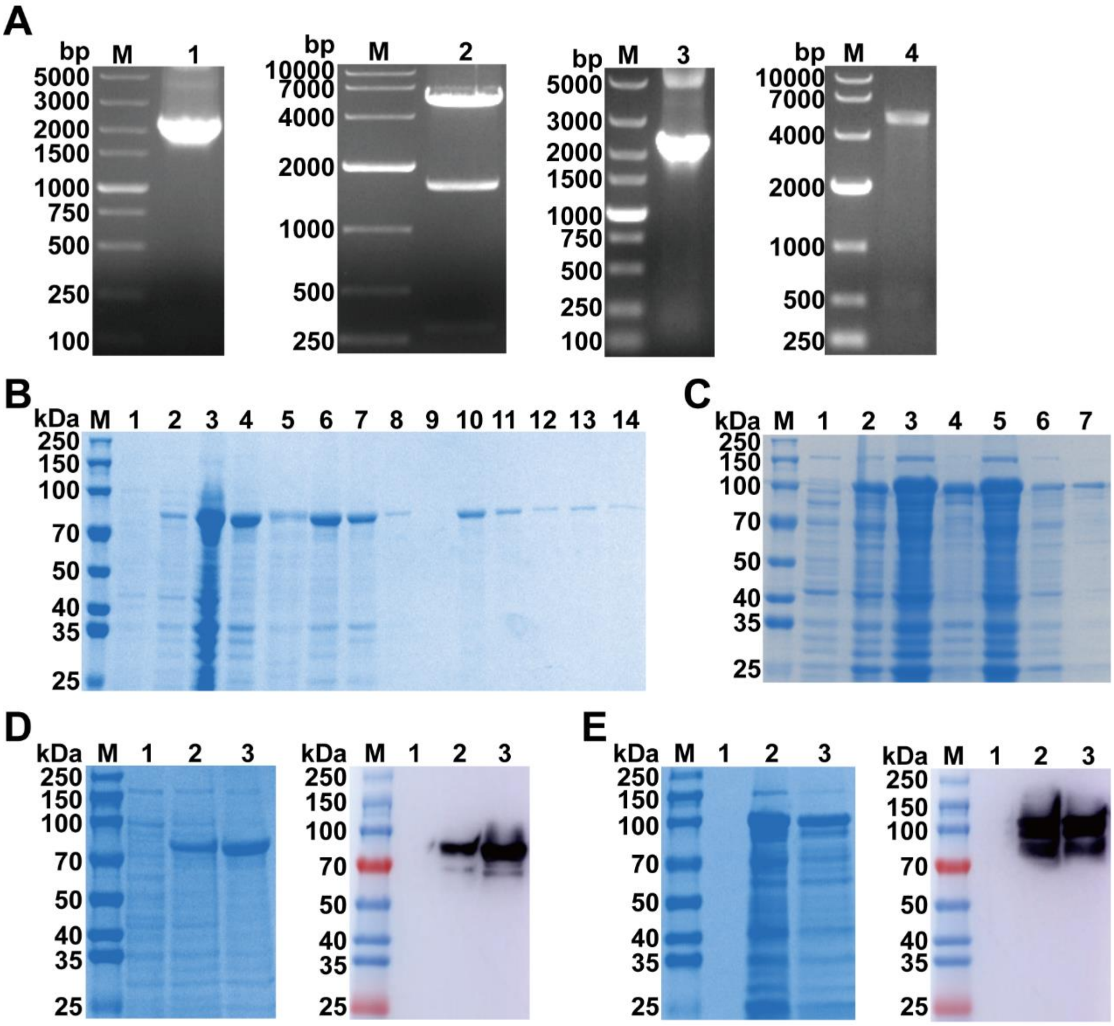
A
bp
M
1
5000
3000
2000
1500
1000
750
500
250
100
2
10000
7000
4000
2000
1000
500
250
3
5000
3000
2000
1500
1000
750
500
250
100
4
10000
7000
4000
2000
1000
500
250
B
kDa
M
1
2
3
4
5
6
7
8
9
10
11
12
13
14
250
150
100
70
50
40
35
25
C
kDa
M
1
2
3
4
5
6
7
250
150
100
70
50
40
35
25
D
kDa
M
1
2
3
250
150
100
70
50
40
35
25
E
kDa
M
1
2
3
250
150
100
70
50
40
35
25

Supplementary Figure S2. Preparation and characterization of a rabbit-derived pAb to FCV VP1 protein. (A) Schedule of rabbit immunization and sample collection. Schematic flow diagram of rabbit immunization procedure for the preparation of rabbit polyclonal antibody using purified recombinant VP1 protein. (B) FCV anti-VP1 antibody detection. Serum antibody titers of immunized rabbit were detected by ELISA. (C) and (D) Immunoblotting and IFA were used to evaluate the reactivity of rabbit polyclonal antibodies. Immunoblotting analysis showing the specificity of rabbit anti-VP1 polyclonal antibody against VP1 protein (C). IFA demonstrating specific recognition of FCV VP1 protein in infected cells by the rabbit anti-VP1 antibody (D)

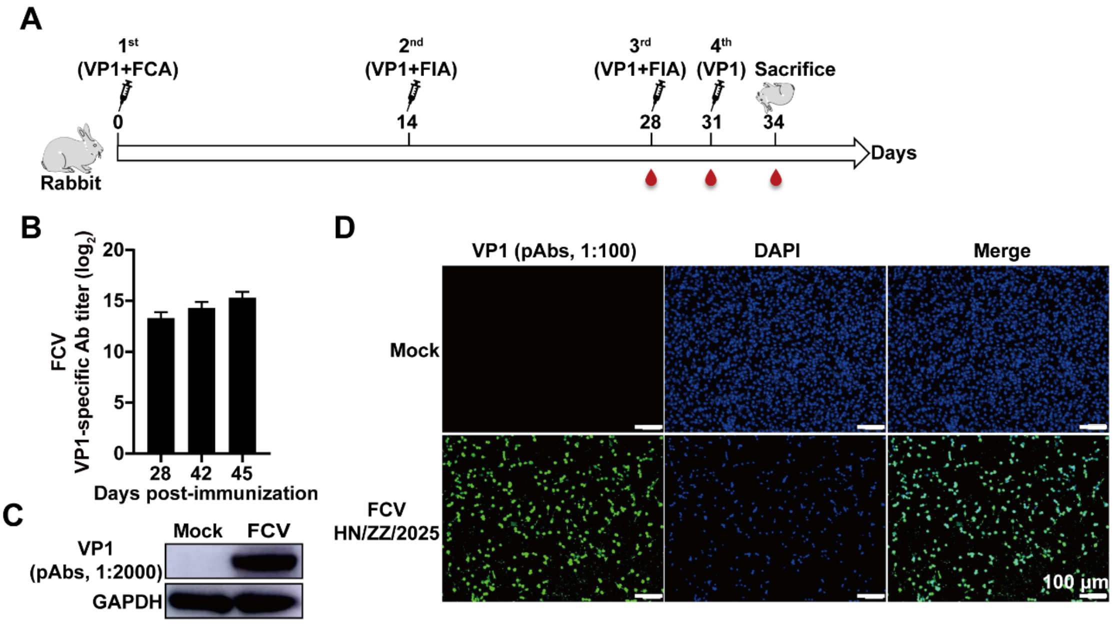

Supplement: Supplementary file 1 [file microorganisms-14-01958-s001.zip › Supplementary Figures S1 and S2.pdf]
