# Supplementary material for: Isolation, Molecular Characterization, and Pathogenicity Evaluation of a Genotype GI Feline Calicivirus Isolate
Source: Microorganisms. 2026 Sep 4;14(9):1958. doi: 10.3390/microorganisms14091958 (PMC13608943; doi:10.3390/microorganisms14091958)
Supplement: Supplementary file 1 [file microorganisms-14-01958-s001.zip › Supplementary Table S1.pdf]

Supplementary Table S1 Identification primer sequences

Primer names	primer sequence 5´-3´	Fragment size
FCV VP1-JD-F	GCAAGTCGCACAGACTGGAT	398 bp
FCV VP1-JD-R	GCCTCCTGACTCTTTTGCTG	
FPV NS1-JD-F	TGTACTTTGCGGGACTTGGT	401 bp
FPV NS1-JD-R	ACATTACCCACAGCTTGTGCT	
FHV TK-JD-F	GACGTGGTGAATTATCAGC	288 bp
FHV TK-JD-R	CAACTAGATTTCCACCAGGA	
FIPV N-JD-F	GCTCTTCCATTGTTGGCTCGTC	198 bp
FIPV N-JD-R	CCGAGGAATTACTGGTCATCGCG	
FBoV NP1-JD-F	ACGTGGGATCAATGTCGTGA	196 bp
FBoV NP1-JD-R	CAGCGTCAGTTACAGGGTCA	
FeAstV ORF1a-JD-F	CTCCGTATCGTACTTGAA	125 bp
FeAstV ORF1a-JD-R	GCCAACACAATAACTGTA	
