# Supplementary material for: Isolation, Molecular Characterization, and Pathogenicity Evaluation of a Genotype GI Feline Calicivirus Isolate
Source: Microorganisms. 2026 Sep 4;14(9):1958. doi: 10.3390/microorganisms14091958 (PMC13608943; doi:10.3390/microorganisms14091958)
Supplement: Supplementary file 1 [file microorganisms-14-01958-s001.zip › Supplementary Table S2.pdf]

Supplementary Table S2 Primer Sequences for FCV genome amplification

Primer names	primer sequence 5´-3´	Fragment size
FCV ORF1-F	ATGTCTCAAACTCTGAGCTTCGTG	5298 bp
FCV ORF1-R	ACATGCTCAAACTTCGAACACATCACAGT	
FCV VP1-F	ATGTGCTCAACCTGCGCTAACGT	2010 bp
FCV VP1-R	TCATAATYTRGTCATTGARCTCC	
FCV VP2-F	ACGTGGGTAAATTGGAGTTTCCCCT	461 bp
FCV VP2-R	GCCCGAAGGGATAVRTGACCAAATT	
